# Supplementary material for: Engineering a 3D Vascularized Adipose Tissue Construct Using a Decellularized Lung Matrix
Source: Biomimetics (Basel). 2021 Sep 18;6(3):52. doi: 10.3390/biomimetics6030052 (PMC8482279; doi:10.3390/biomimetics6030052)
Supplement: Supplementary file 1 [file biomimetics-06-00052-s001.zip › biomimetics-1332452-supplementary.pdf]

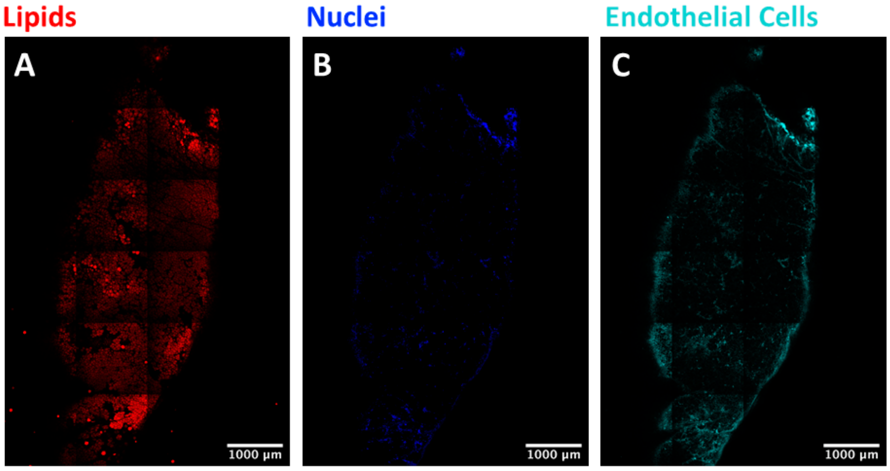

Figure S1. Effective delivery of adipocytes and endothelial cells into the alveolar and vascular compartments of the DLM. Tile scan of entire left lobe showing individual channels (red—adipocytes, teal—endothelial cells, blue—nuclei). Image indicates that adipocytes seeded through the trachea remain in the inner portion of the lobe and are surrounded by the endothelial cells seeded through the vasculature. Scale bar = 1000 µm.
